# Chapter 6: Structural Variation and Medical Genomics

**DOI:** 10.1371/journal.pcbi.1002821

**Published:** 2012-12-27

**Authors:** Benjamin J. Raphael

**Affiliations:** Department of Computer Science and Center for Computational Molecular Biology, Brown University, Providence, Rhode Island, United States of America; Whitehead Institute, United States of America; University of Maryland, Baltimore County, United States of America

## Abstract

Differences between individual human genomes, or between human and cancer genomes, range in scale from single nucleotide variants (SNVs) through intermediate and large-scale duplications, deletions, and rearrangements of genomic segments. The latter class, called structural variants (SVs), have received considerable attention in the past several years as they are a previously under appreciated source of variation in human genomes. Much of this recent attention is the result of the availability of higher-resolution technologies for measuring these variants, including both microarray-based techniques, and more recently, high-throughput DNA sequencing. We describe the genomic technologies and computational techniques currently used to measure SVs, focusing on applications in human and cancer genomics.

**What to Learn in This Chapter**

- Current knowledge about the prevalence of structural variation in human and cancer genomes.
- Strategies for using microarray and high-throughput DNA sequencing technologies to measure structural variation.
- Computational techniques to detect structural variants from DNA sequencing data.

This article is part of the “Translational Bioinformatics” collection for *PLOS Computational Biology*.

## 1. Introduction

The decade since the assembly of the human genome has witnessed dramatic advances in understanding the genetic differences that distinguish individual humans and that are responsible for specific traits. Genome-wide association studies (GWAS) in humans have identified common *germline*, or inherited, DNA variants that are associated with various common human diseases, including diabetes, heart disease, etc. At the same time, cancer genome sequencing studies have cataloged numerous *somatic* mutations that arise during the lifetime of an individual and that drive cancer progression. These successes are ushering in the era of personalized medicine, where treatment for a disease is tailored to the genetic characteristics of the individual.

Despite this progress, significant hurdles remain in achieving a comprehensive understanding of the genetic basis of human traits and disease. The germline variants discovered by GWAS thus far explain only a small fraction of the heritability of many traits, and this “missing heritability” gap [1] is a major bottleneck for future GWAS. The somatic mutations measured in cancer genomes are very heterogeneous, with relatively few mutations that are shared by large numbers of cancer patients, even those with the same (sub)type of cancer. This mutational heterogeneity complicates efforts to distinguish functional mutations that drive cancer development from random passenger mutations [2].

Comprehensive studies of the genetic basis of disease require the measurement of *all* variants that distinguish individual genomes. Until recently, GWAS focused on the measurement of single nucleotide polymorphisms (SNPs), or single nucleotide differences between individual genomes. In the past few years, it has become clear that germline variants occupy a continuum of scales ranging from SNPs to larger structural variants (SVs) – duplications, deletions, inversions, and translocations of large (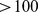 nucleotides) blocks of DNA sequence. Moreover, until recently GWAS focused attention on common SNPs, those whose frequency in the population was at least 5%. This restriction was part of the “common disease, common variant” hypothesis which posits that an appreciable fraction of susceptibility to common diseases results from germline variants that are common in the population. However, this restriction was also dictated by technological limitations, as it was not cost effective to measure all genetic variants in the large number of individual genomes that are necessary to perform a GWAS.

In the past five years, next-generation DNA sequencing technologies became commercially available from companies such as 454, Illumina, Life Technologies, and Complete Genomics. These and other sequencing technologies continue to advance at a breathtaking pace, and consequently the cost of DNA sequencing has declined by several orders of magnitude in the past decade. These technologies provide an unprecedented opportunity to measure all variants; germline and somatic; SNPs and SVs, in both normal and cancer genomes.

In this chapter, we discuss the application of these sequencing technologies in medical genomics, and specifically on the characterization of structural variation.

## 2. Germline and Somatic Structural Variation

Structural variants are important contributors to genome variation and consideration of these variants is necessary for disease association and cancer genetics studies. In this section, we briefly review current knowledge about structural variation in human and cancer genomes.

### 2.1 Germline Structural Variation

Characterizing the DNA sequence differences that distinguish individuals is a major challenge in human genetics. Until a few years ago, the primary focus was to identify single nucleotide polymorphisms (SNPs), and projects such as HapMap [3] provide catalogs of common SNPs in several human populations. Recent whole-genome sequencing and microarray measurements have shown that structural variation, including duplications, deletions, and inversions of large blocks of DNA sequence, is common in the human genome [4]. SVs include both copy number variants – duplications and deletions – that change the number of copies of a segment of the genome, and balanced rearrangements – such as inversions and translocations – that do not alter the copy number of the genome. The Database of Genomic Variants [5] currently (winter 2011) lists apprroximately 66 thousand copy number variants and approximately 900 inversion variants in the human genome, and this number continues to increase. Some of these entries are multiple reports of the same variant due to problems in merging SV predictions across different platforms/technologies (see Section 5 below). Nevertheless, SVs are extensive in human populations.

Germline SVs account for a greater share of the total nucleotide differences between two individual human genomes than SNPs [6]. Copy number variants alone account for approximately 18% of genetic variation in gene expression, having little overlap with variation associated to SNPs [7], and can affect the expression of genes up to 300 kb away from the variant [8]. Both common and rare SVs have recently been linked to several human diseases including autism [9] and schizophrenia [10]. In addition to SVs that cause disease, SVs segregating in a population perturb patterns of linkage disequilibrium and haplotype structure [11]. Thus, it is essential to catalog SVs in order to understand their consequences for human population genetics. Incorrect identification of SVs in samples can lead to spurious genetic associations resulting from the undetected SVs, erroneous merging of distinct variants in different samples, and failure to recognize heterozygosity at a locus.

Finally, structural variants are also present in model organisms such as mouse and fruit fly. Identifying these variants is important for animal models of human diseases.

### 2.2 Somatic Structural Variation and Cancer

Cancer is a disease driven by somatic mutations that accumulate during the lifetime of an individual. The inheritance of mutations by daughter cells during mitosis and selection for advantageous mutations make cancer a “microevolutionary process” [12], [13] within a population of cells. Decades of cytogenetic studies have shown that somatic structural variants are a feature of many cancer genomes. These early studies, particularly in leukemias and lymphoma, identified a number of *recurrent* chromosomal rearrangements that are present in many patients with the same type of cancer. For example, a significant fraction of patients with chronic myelogenous leukemia (CML) exhibit a translocation between chromosomes 9 and 22. The breakpoints of this translocation lie in two genes, BCR and ABL, and the translocation results in the BCR-ABL fusion gene that is directly implicated in the development of this cancer. In addition to fusion genes, somatic SVs can also lead to altered expression of oncogenes and tumor suppressor genes due to both genetic and epigenetic mechanisms [14]. For example, in Burkitt's lymphoma, a translocation activates the MYC oncogene by fusing it with a strong promoter.

In solid tumors, the situation is more complicated. Many solid tumors have genomes that are extensively rearranged compared to the normal healthy genome from which they were derived [14]. These highly rearranged genomes are thought to be a product of genome instability resulting from mutations in the DNA repair machinery. This complex organization of cancer genomes obscures functional driver SVs in a background of passenger mutations. However, with the availability of higher-resolution genomics technologies, recurrent fusion genes are also being found in solid tumors, such as prostate [15] and lung cancers [16]. These results suggest that additional events remain to be discovered [17]. Next-generation DNA sequencing technologies provide the opportunity to reconstruct the organization of cancer genomes at single nucleotide resolution [18], [19]. Projects including The Cancer Genome Atlas (TCGA) (http://cancergenome.nih.gov) and International Cancer Genome Consortium (ICGC) are using these technologies to measure somatic mutations in thousands of cancer genomes [20].

### 2.3 Mechanisms of Structural Variation

As additional genetic and somatic structural variants are characterized, there is increasing opportunity to characterize the mechanisms that produce these variants. A distinguishing feature of the different mechanisms is the amount of sequence similarity, or homology, at the breakpoints of the structural variant. One extreme is little or no sequence similarity. These variants are thought to result from random (or near random) double-stranded breaks in DNA. These breaks might occur due to exposure to external DNA damaging agents. For example, ultraviolet radiation or various chemotherapy drugs produce double-stranded breaks. Aberrant repair of these breaks result in structural variants. This mechanism is termed non-homologous end-joining (NHEJ) [21], [22].

**Figure 1:**
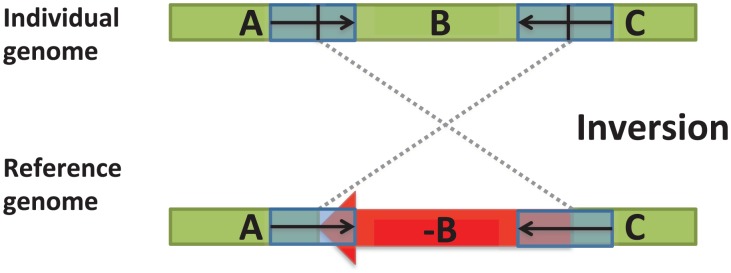
An inversion resulting from non-allelic homologous recombination (NAHR) between two nearly identical segmental duplications (blue boxes) with opposite orientations (arrows). The inversion flips the orientation of the subsequence, or block, 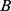 in one genome relative to the other genome.

The opposite extreme is high sequence similarity at the breakpoints. This mechanism is termed non-allelic homologous recombination (NAHR). This mechanism is similar to the normal biological process of homologous recombination that occurs during meiosis and exchanges DNA between two homologous chromosomes. But as the name states, NAHR is a rearrangement that occurs between homologous sequences that are not the same allele on homologous chromosomes. Rather NAHR occurs between repetitive sequences on the genome (Figure 1) [23]–[25]. The human genome contains numerous repetitive sequences ranging from *Alu* elements of 300 bp to segmental duplications, also called low copy repeats, of tens to hundreds of kbp [26]. Thus, there are numerous substrates for NAHR in the human genome, and not surprisingly numerous reported structural variants that result from NAHR. For example, the 1000 Genomes Project, a large NIH project to survey all classes of variation – SNPs through SV – in 1000 human genomes recently reported that approximately 23% of deletions were a result of NAHR [27]. Importantly, due to technical limitations in discovering NAHR-mediated SVs (see below), this percentage may be an underestimate.

There are other mechanisms for the formation of SVs. The division between homology mediated and non-homologous mechanisms may not be so strict. NHEJ events sometimes have some degree of microhomology (e.g. 2–25 bp of similarity) at their breakpoints. Other mechanisms such as fork stalling and template switching (FoSTeS) have also been proposed. Some of these are reviewed in [28]. Finally, the relative contribution of each of these mechanisms in generating germline SVs versus somatic SVs remains an active area of investigation, with conflicting reports about the importance of repetitive sequences in somatic structural variants found in cancer genomes [21], [22], [24], [25], [29].

## 3. Technologies for Measurement of Structural Variation

Structural variants vary widely in size and complexity, ranging from insertions/deletions of hundreds of nucleotides to large scale chromosomal rearrangements. Large structural variants can be visualized directly on chromosomes, through cytogenetic techniques such as chromosome painting, spectral karyotyping (SKY), or fluorescent in situ hybridization (FISH). In fact, Sturtevant and Dobzhansky studied inversion polymorphisms in *Drosophila* in the 1920's – well before the modern genomics era. However, SVs that are too small to be directly observed on chromosomes are generally more difficult to detect and to characterize than single nucleotide polymorphisms (SNPs). Much of the recent excitement surrounding structural variation stems from improvements in genomics technologies that allow more complete measurements of SVs of all types. These include microarrays and more recently next-generation DNA sequencing technologies. In this section, we briefly describe these technologies.

### 3.1 Microarrays

The first genome-wide surveys of SVs in the human genome in 2004 utilized microarray-based techniques such as array comparative genomic hybridization (aCGH). In aCGH, differentially fluorescently labeled DNA from an *individual*, or *test*, genome and a *reference* genome are hybridized to an array of genomic probes derived from the reference genome. Measurements of test∶reference fluorescence ratio, called the copy number ratio, at each probe identifies locations of the test genome that are present in higher or lower copy in the reference genome. Microarrays containing hundreds of thousands of probes are available, and thus one obtains copy number ratios at hundreds of thousands of locations. Since individual copy number ratios are subject to various types of experimental error, computational techniques are needed to analyze aCGH data. For further details about aCGH and aCGH analysis, see [30].

aCGH is equally applicable for measurement of germline SVs in normal genomes and somatic SVs in cancer genomes. In fact, aCGH was originally developed for cancer genomics applications. aCGH is now very affordable making it possible to detect copy number variants in large numbers of genomes at reasonable cost. However, aCGH has two important limitations. First, because aCGH measures only differences in the number of copies of a genomic region between a test and reference genome, aCGH detects only copy number variants. Thus, aCGH is blind to copy-neutral, or balanced, variants such as inversions, or reciprocal translocations. Moreover, aCGH requires that the genomic probes from the reference genome lie in non-repetitive regions, making it difficult to detect SVs with breakpoints in repetitive regions, such as NAHR events or the insertion/deletion of repetitive sequences.

### 3.2 Next-generation DNA Sequencing Technologies

DNA sequencing technology has advanced dramatically in recent years, and several “next-generation” DNA sequencing technologies from companies such as Illumina, ABI, and 454 have significantly lowered the cost of sequencing DNA. However, these technologies, and the Sanger sequencing technique they are replacing, are severely limited in the length of a DNA molecule that can be sequenced. Present sequencing technologies produce short sequences of DNA, called reads, that range from 25–1000 nucleotides, or base pairs (bp), with the upper end of this range requiring technologies (e.g. Sanger and 454) that are considerably more expensive. Much of the recent excitement in DNA sequencing has been in *short read* DNA sequencers (e.g.llumina Genome Analyzer, Life Technologies SOLiD and Ion Torrent) that yield reads of only 25–150 nucleotides. These reads are much shorter than the one to two hundred million bp of a typical human chromosome. However, the large number of reads that are produced (hundreds of millions), results in a cost per nucleotide that is several orders of magnitude lower than Sanger sequencing.

Many DNA sequencing technologies employ a paired end, or mate pair, sequencing protocol to increase the effective read length. In this protocol two reads are generated from opposite ends of a longer DNA fragment, or insert. With earlier Sanger sequencing protocols, the sizes of these DNA fragments were dictated by the cloning vector that was used. Fragment, or insert, sizes of 2 kb–150 kb could be obtained by cloning into bacterial plasmids or bacterial artificial chromosomes (BACs). With next-generation technologies, a variety of techniques have been employed to generate paired reads. At present, the most efficient and effective techniques produce paired reads from fragments of only a few hundred bp, although fragments of 2–3 kb are available. Thus, next-generation sequencing technologies have both limited read lengths and limited insert sizes compared to Sanger sequencing.

**Figure 2:**
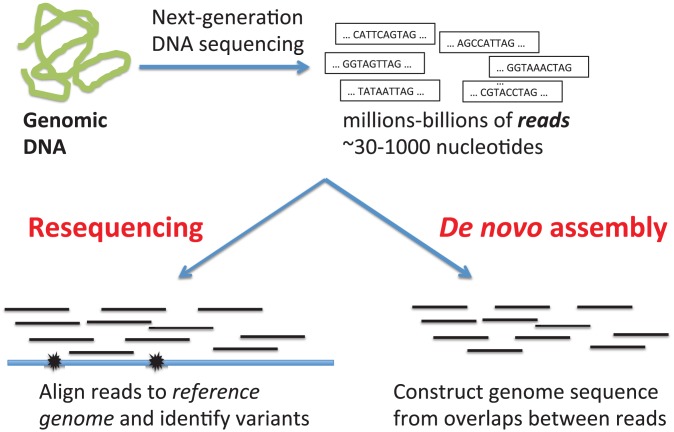
Two major approaches to detect structural variants in an individual genome from next-generation sequencing data are *de novo* assembly and resequencing. In *de novo* assembly, the individual genome sequence is constructed by examining overlaps between reads. In resequencing approaches, reads from the individual genome are aligned to a closely related reference genome. Examination of the resulting alignments reveals differences between the individual genome and the reference genome.

There are two approaches to detecting SVs from next-generation DNA sequencing data (Figure 2). The first is *de novo* assembly. In this approach, sophisticated algorithms are used to reconstruct the genome sequence from overlaps between reads. The assembled genome sequence is then compared to the reference genome, or the assembled genomes of other individuals, to identify all types of variants. If the genome sequence is successfully assembled, this approach is the best for characterization of SVs. Unfortunately, assembling a human genome *de novo* – i.e. with no prior information – of sufficient quality for structural variation studies remains difficult with limited read lengths. Currently, human genome assemblies are highly fragmented, consisting of tens-hundreds of thousands of contigs, intermediate sized sequences of thousands to tens of thousands of nucleotides. Moreover, the associations between some structural variants and repetitive sequences implies that assemblies of *finished* (not *draft* quality) are necessary for comprehensive coverage of structural variation. Improving *de novo* assembly is a very active research area (see [31]), but human genome assemblies of high enough quality for SV studies remain out of reach for inexpensive short-read technologies.

The second approach to detect SVs in next-generation DNA sequencing data is a “resequencing” approach that leverages the extensive finishing efforts undertaken in the Human Genome Project. In a resequencing approach, one finds differences between an individual genome and a closely related reference genome whose sequence is known by aligning reads from the individual genome to the reference genome. Differences (variants) between the genomes correspond to differences between the aligned reads and the reference sequence. In the next section, we describe how to predict SVs using a resequencing approach.

### 3.3 New DNA Sequencing Technologies

Many of the challenges in reliable measurement of SVs described above are related to limitations in sequencing technologies. In particular, SVs with breakpoints in highly-repetitive sequences are beyond the abilities of current technologies. New “third-generation” and single-molecule technologies promise additional advantages for structural variation discovery. These advantages include longer read lengths, easier sample preparation, lower input DNA requirements, and higher throughput. For example, Pacific Biosciences recently released their Single-Molecule Real Time (SMRT) sequencing, a technology that measures in real time the incorporation of nucleotides by a single DNA polymerase molecule immobilized in a nanopore [32].

**Figure 3:**
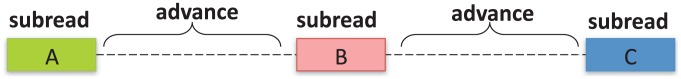
A strobe with 3 subreads.

One application of this technology is *strobe sequencing*. A strobe read, or strobe, consists of multiple subreads from a single contiguous molecule of DNA. These subreads are separated by a number of “dark” nucleotides (called advances), whose identity is unknown (Figure 3). Thus far, Pacific Biosciences has demonstrated strobes of lengths up to 20 kb with 2–4 subreads each of 50–400 bp. Additional improvements are expected as technology matures. Strobes generalize the concept of paired reads by including more than two reads from a single DNA fragment. Strobes provide long-range sequence information with low input DNA requirements, a feature missing from current sequencing technologies. This additional information is useful for detection and *de novo* assembly of complex SV that lie in highly repetitive regions, or contain multiple breakpoints in a small region. However, the advantages of strobes are reduced by higher single-nucleotide error rates. Thus, realizing the advantages of strobes requires new algorithms that exploit information from multiple, spaced subreads to overcome high single-nucleotide error rates [33].

Sequencing technologies continues its rapid development. Improvements in the chemistry, imaging, and manufacture of existing technologies are increasing their read lengths, insert lengths, and throughput. Additional sequencing technologies are under active development. Nanopore-based technologies that directly read the nucleotides of long molecules of DNA hold promise for a dramatic shift in DNA sequencing where extremely long reads (tens of kb) are generated, making both *de novo* assembly and variant detection by resequencing straightforward problems.

## 4. Resequencing Strategies for Structural Variation

A resequencing strategy predicts SVs by alignments of sequence reads to the reference genome. There are two main steps in any resequencing strategy: (1) alignments of reads; (2) prediction of SVs from alignments. Resequencing approaches are straightforward in principle, but in practice sensitive and specific detection of structural variation in human genomes is notoriously difficult [34], [35]. While some types of SVs are easy to detect with next-generation sequencing technologies, other complex SVs are refractory to detection. This is due to both technological limitations and biological features of SVs. DNA sequencing technologies produce reads with sequencing errors, have limited read lengths and insert sizes, and have other sampling biases (e.g. in GC-rich regions). Biologically, human SVs are: (i) enriched for repetitive sequences near their breakpoints [23]; (ii) may overlap, have multiple states or complex architectures; and (iii) recurrent (but not identical) variants may exist at the same locus [36], [37]. These properties mean that the alignment of reads to the reference genome and the prediction of SVs from these alignments is not always an easy task. Algorithms are required to make highly *sensitive* and *specific* predictions of SVs.

In this section we review the main issues in predicting SVs using a resequencing approach. We begin with read alignment. Then we describe the three major approaches that are used to identify structural variants from aligned reads: (i) split reads; (ii) depth of coverage analysis; and (iii) paired-end mapping.

### 4.1 Read Alignment

Alignment of reads to a reference genome is a special case of sequence alignment, one of the most researched problems in bioinformatics. However, the specialized task of aligning millions-billions of individual short reads led to the development of new software programs tailored to this task, such as Maq, BWA, Bowtie/Bowtie2, BFAST, mrsFAST, etc. [38]–[43]. A key decision in read alignment for SV detection is whether to consider only reads with a single, best alignment to the reference genome, or to also include reads with multiple high-quality alignments. Some read alignment programs will output only a single alignment for each read, in some cases choosing an alignment randomly if there are multiple alignments of equal score. If one uses only reads with a unique alignment, then there is limited power to detect SVs whose breakpoints lie in repetitive regions, such as SVs resulting from NAHR. On the other hand, if one allows reads whose alignment is ambiguous, then the problem of SV prediction requires an algorithm to distinguish among the multiple possible alignments for each read. Many SV prediction algorithms analyze only unique alignments, although several recent algorithms use ambiguous alignments. A few of these are noted below.

### 4.2 Split Reads

**Figure 4:**
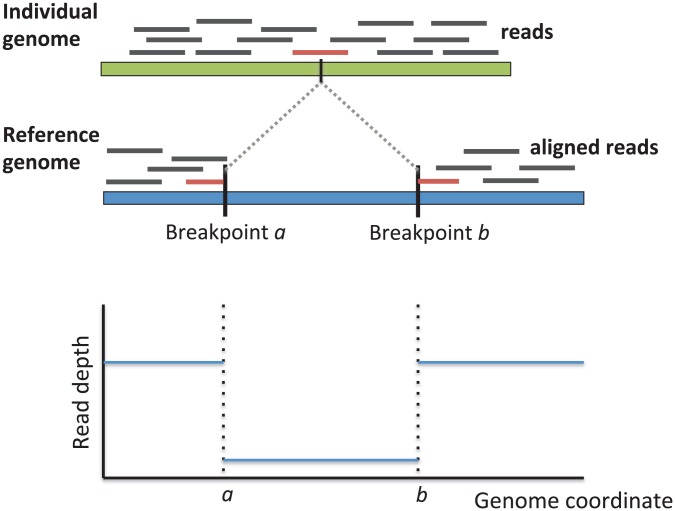
Identification of a deletion in an individual genome by split read analysis (middle), and by depth of coverage analysis (bottom).

A direct approach to detect structural variants from aligned reads is to identify reads whose alignments to the reference genome are in two parts. These so called split reads contain the breakpoint of the structural variant (Figure 4). To reduce false positive predictions of structural variants, one requires the presence of multiple split reads sharing the same breakpoint. Because the two parts of a split read align independently to the reference genome, these alignments must be long enough to be aligned uniquely (or with little ambiguity) to the reference. Thus, split read analysis is a feasible strategy only when the reads are sufficiently long. For example, if one has a 36 bp read containing the breakpoint of an SV at its midpoint, one must align the two 18 bp halves of the read to the reference genome. Finding unique alignments of an 18 bp sequence is often not possible. There are no reports of successful prediction of structural variants from split reads alone using next generation DNA sequencing reads less that 50 bp in length. Instead, split read methods have been proposed that use paired reads, and require that one read in the pair has a full length alignment to the reference. This alignment of the read from one end of the fragment is used to anchor the search for alignments of the other split read of the fragment [44]–[46].

### 4.3 Depth of Coverage

 Depth of coverage (also called read depth) analysis detects differences in the number of reads that align to intervals in the reference genome. Assuming that reads are sampled uniformly from the genome sequence, the number of reads that contain a given nucleotide of the reference is, on average, 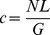, where 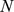 is the number of reads, 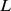 is the length of each read, and 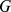 is the length of the genome. This is the Lander-Waterman model, and the parameter 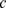, called the coverage, is a key parameter in a sequencing experience. For example, recent cancer sequencing projects with Illumina technology have used “30X coverage” which means that the number of reads and length of reads are chosen such that 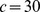.

Now, if the individual genome contained a deletion of a segment of the human reference genome, the coverage of this segment would be reduced by half – if the deletion was heterozygous – or reduced to zero – if the deletion was homozygous (Figure 4). Similarly, if an interval of the reference genome was duplicated, or amplified, in the individual genome, the coverage of this interval would increase in proportion to the number of copies. Thus, the observed coverage of an interval of the reference genome, the depth of coverage, gives an indication of the number of copies of this interval in the individual genome. Of course, there are numerous additional factors to consider beyond this simple analysis. For example, since reads are sampled at random from the genome, coverage is not constant, but rather follows a distribution with mean 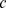. A Poisson distribution is typically used as an approximation to this distribution, although other distributions sometimes provide a better fit to the data. In addition, repetitive sequences in the reference genome and biases in sequencing (e.g. different coverage of GC-rich regions) also affect depth of coverage calculations. Nevertheless, there are several computational methods for depth of coverage analysis [47], [48]. Many of these are largely similar to those used to analyze microarray copy number data.

### 4.4 Paired-end Sequencing and Mapping

**Figure 5:**
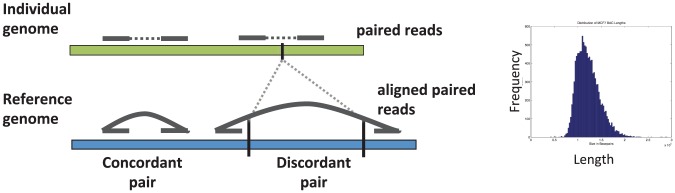
Paired end mapping (PEM). Fragments from an individual genome are sequenced from both ends and the resulting paired reads are aligned to a reference genome. Most paired reads correspond to concordant pairs, where the distance between the alignment of each read agrees with the distribution of fragment lengths (right). The remaining discordant pairs suggest structural variants (here a deletion) that distinguish the individual and reference genomes.

The most common approach for resequencing SVs is paired-end mapping (PEM) (Figure 5). Paired-end mapping was used to identify somatic SVs in cancer genomes [49], [50] and the same idea has been applied to identify germline structural variants [51], [52]. While the early paired-end mapping studies used older clone-based sequencing, paired-end mapping is now possible using various next-generation sequencing technologies.

In PEM, a paired-end sequencing protocol is used to obtain paired reads from opposite ends of a larger DNA fragment, or clone, from a *individual genome*. These paired reads are then aligned to a reference genome. Most paired reads result in concordant pairs where the distance between aligned reads is equal to the fragment length. In contrast, discordant pairs have alignments with abnormal distance or that lie on different chromosomes. These suggest the presence of an SV or a sequencing error. For example, a discordant pair whose distance between alignments is too long suggests a deletion in the individual genome (Figure 5), while a discordant pair whose alignments are on different chromosomes suggests a translocation. Other types of discordant pairs identify inversions, transpositions, or duplications that distinguish the individual genome from the reference genome. Note that in general the length of any particular sequenced fragment is not known. Rather, during the preparation of genomic DNA for sequencing, the DNA is fragmented and fragments are size-selected to an appropriate target length. It is desirable for this size selection to be as strict as possible, so that only fragments near the target length are sequenced. However, in practice the size selection procedure produces fragments whose lengths vary around the target length. Typically, the distribution of fragment lengths is obtained empirically by examining the distances between all aligned paired reads, as most fragments will correspond to a concordant pair (Figure 5).

To distinguish real SVs from sequencing errors, one looks for clusters of discordant pairs that indicate the same SV. Numerous algorithms have been developed to predict SVs by finding clusters of discordant pairs. Early algorithms used only those paired reads whose alignments to the reference genome were non-ambiguous; i.e. there was only a single “best alignment” [53]–[55]. More sophisticated algorithms use paired reads with multiple ambiguous alignments to the reference genome and use a variety of combinatorial and statistical techniques to select among these alignments [56]–[58]. Finally, some approaches model the fact that the human genome is diploid to avoid making inconsistent structural variant predictions [59].

**Figure 6:**
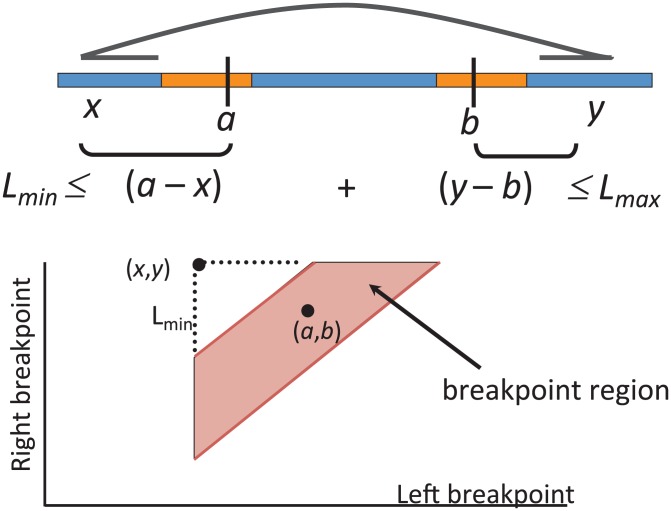
(Top) A discordant pair (arc) indicates a deletion with unknown breakpoints 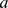 and 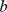 located in orange blocks. Positions 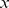, 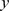 and the minimum 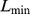 and maximum 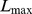 length of end-sequenced fragments constrain breakpoints 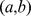 to lie within the indicated orange blocks, and are governed by the indicated linear inequalities. (Bottom) A polygon in 2D genome space expresses the linear dependency between breakpoints 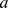 and 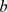 and records the uncertainty in the location of the breakpoints.

All of the approaches above rely on predicting structural variants that are supported by multiple paired reads. Some, but not all, of them are careful when determining whether a group of paired reads genuinely support the same variant. We illustrate the issue here using the Geometric Analysis of Structural Variants (GASV) method of [55]. A key feature of GASV is that it records both the information that the paired reads reveal about the boundaries (breakpoints) of the structural variant *and the uncertainty associated with this measurement*. Most types of SV, including deletions, inversions, and translocations have two breakpoints 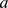 and 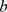 where the reference genome is cut. The segments adjacent to these coordinates are then pasted together in a way that is particular to the type of SV. For example, a deletion is defined by coordinates 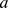 and 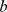 in the reference genome such that the nucleotide at position 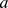 is joined to the nucleotide at position 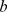 in the individual genome (Figure 6). Note that this is a simplification of the underlying biology, as there are sometimes small insertions or deletions at breakpoints, but these small changes have limited effect on the analysis of larger structural variants.

Now the discordant pairs that indicate an SV have the property that the locations of the read alignments are near the breakpoints 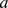 and 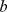. However, a paired read does not give independent information about the breakpoint 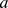 and the breakpoint 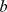. Rather, the breakpoints 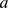 and 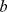 are related by a linear inequality that defines a polygon in 2D genome space called the breakpoint region (Figure 6). For example, suppose that the pair of reads from a single fragment align to the same chromosome of the reference genome such that the read with lower coordinate starts at position 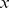 in the reference and the read with higher coordinate ends at position 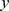 in the reference. (For simplicity, we ignore the fact that the sequence of a read can align to either strand (forward or reverse) of the reference genome. The strand of an alignment gives additional information about the location of the breakpoint. See [55] for further details.) If the sequenced fragment has length 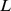 then the breakpoints 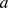 and 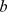 satisfy the equation 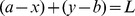. As described above, the size of any particular fragment is typically unknown. Rather, one defines a minimum size 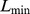 and maximum size 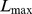 of a sequenced fragment, perhaps according to the empirical fragment length distribution. Thus, we have the inequality
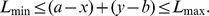
This equation defines the unknown breakpoints 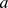 and 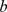 in terms of the known coordinates 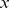 and 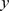 of the aligned reads and the length of sequenced fragments. The pairs of breakpoints 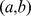 that satisfy this equation form a polygon (specifically a trapezoid) in two-dimensional genome space. We define the breakpoint region 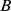 of discordant pair 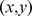 to be the breakpoints 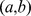 satisfying the above equation.

This geometric representation provides a principled way to combine information across multiple paired-reads: multiple paired-reads indicate the same variant if their corresponding breakpoint regions intersect. The geometric representation also provides precise breakpoint localization by multiple paired reads; separates multiple measurements of the same variant from measurements of nearby or overlapping variants; and facilitates robust comparisons across multiple samples and measurement technologies. Finally, the approach is computationally efficient as it relies on computational geometry algorithms for polygon intersection. These scale to millions of discordant pairs that result from next-generation sequencing platforms.

While the algorithms above consider many of the issues in prediction of structural variants, there remains room for improvement. Most notably, many algorithms still use only one of the possible signals of structural variants: read depth, split reads, or paired reads. Improvements in specificity are likely possible by integrating these multiple signals into a single prediction algorithm [60].

## 5. Representation of Structural Variants

Next generation DNA sequencing technologies are dramatically reducing the cost of sequence-based surveys of structural variants, while oligonucleotide aCGH techniques are now used in studies profiling tens of thousands of genomes. Large projects like the 1000 Genomes Project and The Cancer Genome Atlas (TCGA) are performing paired-end sequencing and aCGH of many human genomes, and matched tumor and normal genomes, respectively. At the same time, smaller or single investigator projects are using a variety of paired-end sequencing approaches and/or microarray-based techniques with different trade-offs in cost-per-sample vs. measurement resolution. Thus, in the near future there will be an enormous number of measurements of SVs, but using a wide range of technologies of varying resolution, sensitivity, and specificity. This diversity of approaches will likely continue for some time as investigators explore tradeoffs between the cost of measuring variants in one sample with high confidence versus surveying variants in many samples with lower confidence per sample. For example, in cancer genome studies the goal of finding recurrent mutations demands the survey of many genomes and thus large sample sizes might be preferred over high coverage sequencing of one sample.

The problem of comparing variants across samples and/or measurement platforms is less studied than the problem of detecting variants in a single sample. Standard practice remains to use heuristics that merge predicted structural variants into the same variant in they overlap by a significant fraction (e.g. 50–70%) on the reference genome. For example, the Database of Genomic Variations (DGV) [5], arguably the most comprehensive repository of measured human structural variants, merges structural variant predictions whose coordinates overlap by 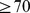
% on the reference genome. Such heuristics are typically the only approach available to databases of human structural variants because many early studies did not report information on the uncertainty (i.e. “error bars”) in the boundaries (breakpoints) of the variant. This situation makes it difficult to explicitly separate multiple measurements of the same variant from measurements of nearby variants or overlapping variants. This situation is now improving, and more recent software records both the information that the measurement reveals about the breakpoints of the structural variant and the uncertainty associated with this measurement. Software that uses this uncertainty to classify and compare SVs across samples and measurement platforms is also now available [55]. Such precision provides increased confidence in associations between a structural variant and a disease, helps separate germline from somatic structural variants in cancer genome sequencing projects, and aids in the study of rare recurrent variants that might occur on a variety of genetic backgrounds.

## 6. Challenges for Cancer Genomics Studies

The study of somatic structural variation in cancer genomes presents additional challenges beyond those described above for generic resequencing approaches. First, most cancer genomes are aneuploid, meaning that the number of copies of regions of the genome are variable, due to duplications and deletions of segments of the normal genome. High-resolution reconstructions of cancer genomes by paired read sequencing showed that many rearrangements were too small to be detected by cytogenetics, and identified highly rearranged genomic loci that encompass a complex intertwining of rearrangement and duplication [21], [29], [49], [50], [61]–[63]. Such highly rearranged loci are hypothesized to result from genome instability caused by defective DNA repair in cancer cells, or from external DNA damage. An extreme example is the phenomenon of chromothripsis that results from massive, simultaneous breakage and aberrant repair of many genomic loci [64]. Identifying all of the SVs and thereby reconstructing the organization of cancer genomes can suggest that certain regions of the genome are selected for their pathogenetic properties, and also lend insight into the mechanisms of genome instability in tumors [14].

**Figure 7:**
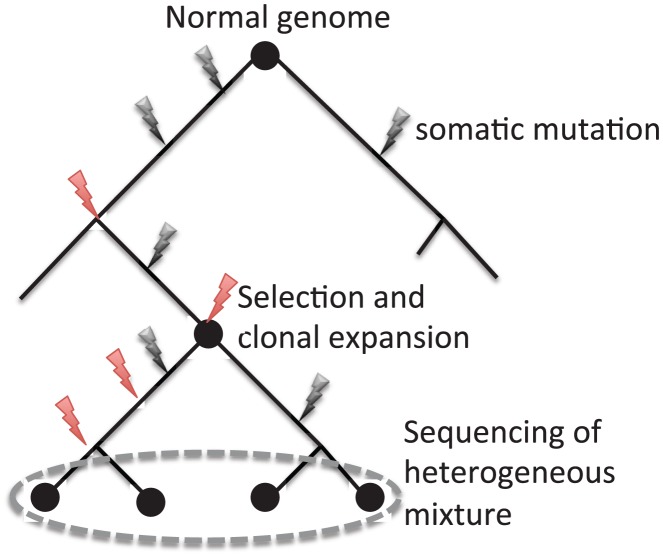
Mutation, selection, and clonal expansion in tumor development leads to genomic heterogeneity between cells in a tumor. Current DNA sequencing approaches sequence DNA from many cells and thus result in a heterogenous mixture of mutations, with varying numbers of both passenger mutations (black) and driver mutations (red).

A second challenge is that cancer tissues are a heterogeneous mixture of cells with possibly different numbers of mutations. This heterogeneity includes admixture between normal and cancer cells, as well as subpopulations of tumor cells. Some of these subpopulations might contain important driver mutations, or drug resistance mutations. Because of the amount of DNA required for current sequencing technologies, most cancer genome sequencing studies do not sequence single tumor cells but rather sequence a mixture of cells (Figure 7). Since the signal for detecting variants is proportional to the number of cells in the mixture that contain the variant, presence of normal cells will reduce the power to detect somatic mutations. Further, the ability to detect mutations that are rare in the tumor cell population will be even lower. Eventually, whole genome sequencing of single cells will provide fascinating datasets to study cancer genome evolution, with some recent hints of the discoveries to come in [65].

## 7. Future Prospects

This chapter described the challenges in identification and characterization of structural variants. With further improvements in sequencing technologies and algorithms over the next few years, it will be possible to systematically measure nearly all but the most complex variants in an individual genome. The most difficult cases, such as variants mediated by homologous recombination between nearly identical sequences, might remain inaccesible until significantly different types of DNA sequencing technologies become available. Nevertheless, the fact that systematic identification of nearly all germline and somatic structural variants in an individual genome is now possible will enable further progress in human and cancer genetics.

For genetic association studies, having complete lists of germline variants from many individuals means that unexplained heritability for a trait cannot readily be blamed on lack of measurement of genetic information. Unfortunately, this does not necessarily imply that finding the genetic basis for specifc traits will become easy. There remain other challenges, including the possiblity that combinations of variants, interactions between genetic and environmental factors, or other epigenetic mechanisms, may contribute to phenotype. See [66] in this collection for further discussion of these issues. Finally, translating genetic information about susceptibility to a disease or efficacy of particular treatments into improved medical outcomes will require additional work.

The opportunities and challenges are similar in cancer genetics. Systematic measurement of all somatic mutations will yield information that might guide treatments, and eventually lead to personalized oncology. Current cancer treatments are limited by the non-specificity of most cancer drugs and by the fact that cancer cells can evolve resistance to single drug treatments. Tailoring of treatment to the particular genetic mutations in a tumor promises to revolutionize cancer therapy. There are already several examples of such personalized treatments including the drug Gleevec that targets the BCR-ABL fusion gene in chronic myelogenous leukemia (CML) and Iressa that targets the EGFR gene in lung cancer. Discovery of additional cancer-specific drug targets requires not only technologies to globally survey somatic mutations in cancer genomes, but also techniques (experimental and/or computational) to classify the subset of variants that are functional, and then the further subset of these functional variants that are druggable.

The sequencing technologies and algorithms described in this chapter are laying the foundation for personalized medicine, but much work remains to translate the information revealed by genome sequencing into improved clinical practice.

## 8. Exercises

1. Consider the chromosomal inversion in Figure 1. What signals in next-generation sequencing data can be used to detect a chromosomal inversion?
2. The human genome is diploid with two copies, maternal and paternal, of each chromosome. What constraints does this place on prediction of germline structural variants?

Answers to the Exercises can be found in Text S1.

**Further Reading**

- Alkan C, Coe BP, Eichler EE (2011) Genome structural variation discovery and genotyping. Nat Rev Genet 12: 363–376.
- Mardis ER (2012) Genome sequencing and cancer. Curr Opin Genet and Dev 22: 245–250.
- Meyerson M, Gabriel S, Getz G (2010) Advances in understanding cancer genomes through second-generation sequencing. Nature Reviews Genetics 11: 685–696.
- Medvedev P, Stanciu M, Brudno M (2009) Computational methods for discovering structural variation with next-generation sequencing. Nat Methods 6: 13–20.
- Sindi S, Helman E, Bashir A, Raphael BJ (2009) A geometric approach for classification and comparison of structural variants. Bioinformatics 25: i222–i230.
- Stratton MR (2011) Exploring the genomes of cancer cells: progress and promise. Science 331: 1553–1558.

## Supporting Information

Text S1 Answers to Exercises.
(PDF)
