# Supplementary material for: Chapter 6: Structural Variation and Medical Genomics
Source: PLoS Comput Biol. 2012 Dec 27;8(12):e1002821. doi: 10.1371/journal.pcbi.1002821 (PMC3531322; doi:10.1371/journal.pcbi.1002821)
Supplement: Text S1 — Answers to Exercises. (PDF) [file pcbi.1002821.s001.pdf]

Structural Variation and Medical Genomics

Benjamin J. Raphael[1]

[1]Department of Computer Science and Center for Computational Molecular Biology, Brown University, Providence, RI, USA

Exercises (With Answers)

(1) Consider the chromosomal inversion in Figure 1. What signals in next-generation sequencing data can be used to detect a chromosomal inversion?

Answer: Split reads or paired-end mapping. Read depth cannot be used because the inversion does not alter the number of copies of a region in the reference genome.

(2) The human genome is diploid with two copies, maternal and paternal, of each chromosome. What constraints does this place on prediction of germline structural variants?

Answer: Predicted structural variants must satisfy the constraint that outside of repetitive sequences in the reference genome, there are at most two copies of each locus. For example there cannot be three overlapping deletions of the same region, or a inversion whose breakpoints lie in a homozygous deletion.
